# The Role of Exhaled Hydrogen Sulfide in the Diagnosis of Colorectal Adenoma

**DOI:** 10.1155/2021/8046368

**Published:** 2021-11-30

**Authors:** Nian Liu, Yujen Tseng, Huilu Zhang, Jian Chen

**Affiliations:** ^1^Sunvou Medical Electronics Company, Wuxi, China; ^2^Department of Gastroenterology, Huashan Hospital of Fudan University, Shanghai, China

## Abstract

**Purpose:**

Exhaled determination can detect metabolite hydrogen sulfide in the intestine. We aim to analyze the predictive value of hydrogen sulfide in the diagnosis of colorectal adenoma.

**Methods:**

We recruited seventy patients diagnosed with colorectal adenoma as the observation group and sixty-six healthy subjects as the control group. The colorectal adenoma was diagnosed by colonoscopy at the Endoscopy Center of Huashan Hospital affiliated to Fudan University from June 2018 to November 2019. Exhaled gas was collected through the nose and mouth, respectively, and hydrogen sulfide in exhaled gas was determined according to the manufacturer's instructions.

**Results:**

Receiver operating characteristic (ROC) curve was analyzed based on the exhaled data of the observation group and the control group. The ROC curve showed an area under ROC curve (AUC) 0.724 for nasal exhaled H_2_S, which had a diagnostic value. When nasal exhaled H_2_S was >13.3 part per billion (ppb), the sensitivity and the specificity of predicting colorectal adenoma were 57% and 78%, respectively. The exhaled H_2_S of the observation group was significantly different from that of the control group. The AUC value was 0.716 as a prognostic factor of colorectal adenoma. As exhaled H_2_S was >28.8 ppb, the sensitivity and the specificity of predicting colorectal adenoma were 63% and 77%, respectively.

**Conclusion:**

Exhaled and nasal H_2_S determination has a predictive value for colorectal adenoma as a novel and noninvasive method. Therefore, it is worth conducting more research to analyze exhaled and nasal H_2_S.

## 1. Introduction

Colorectal cancer (CRC) incidence ranks the third in the world and ranks the fourth in China among malignant diseases [1, 2]. The high incidence of CRC imposes a considerable economic and humanistic burden on the society and patients. 70%–90% of CRC is developed from colorectal adenoma (CRA) [3]. The synthesis and metabolism of signaling molecule-H_2_S is one of the clinical manifestations that provide a theoretical basis for CRA/CRC screening and detection in the progression from CRA to CRC. There are two main sources of H_2_S in the body. H_2_S in the tissue and cells is synthesized by endogenous enzymes: cystathionine*γ*-lyase (CSE), cystathionine-*β*-synthase (CBS), and 3-mercaptopyruvate sulfurtransferase (3-MST). In addition, H_2_S is produced from cystine by the intestinal bacteria [4, 5]. Yamagishi et al. demonstrated that the amount of H_2_S and its derivative methyl mercaptan in CRC patients is significantly higher than that of normal people in the study of digestive tract gas analysis [6]. As a gas molecule, H_2_S can diffuse into the bloodstream and can be exhaled along with expiratory movements. Therefore, H_2_S has the potential to become a biomarker for screening out colon tumors as a simple and noninvasive method [7].

How to screen out adenoma early and interdict it efficiently is an effective way of reducing incidence and mortality of colorectal cancer [8]. Traditional detecting methods including endoscopy, blood test, and stool DNA testing are invasive, expensive, and complicated to implement. As a result, there is an urgent need to look for a more convenient, accurate, and easy-to-carryout method for clinical practice. Gastrointestinal gas analysis is one of the optimistic methods to develop, while the gas-collecting technique is not easy in the antecedent research (anal exhaust and fecal fermentation), and the technique of gas analysis is complex (near edge X-ray absorption fine structure (NEXAFS) spectra and spectrophotometric determination of gas after solution preparation) [6, 9]. Thus, a better gas-determination method is required. For the above reasons, our study determines concentration of nasal and exhaled H_2_S by electrochemical sensors to predict the occurrence of CRA. We hope that exhaled H_2_S determination could provide scientists and researchers some new insight into early screening and warning of CRC.

## 2. Study Design and Methodology

### 2.1. Study Design

70 patients diagnosed with CRA were enrolled as the observation group and 66 healthy subjects without organic lesions were selected as the control group. The diagnosis of CRA was made via colonoscopy screening at the Endoscopy Center of Huashan Hospital Affiliated to Fudan University from June 2018 to November 2019. Inclusion criteria: (1) age 18–80 y old, normal cognitive function, and able to complete exhaled determination; (2) not take any antibiotics or probiotics within 2 weeks before enrollment. Exclusion criteria: (1) take gastrointestinal motility drugs, acid suppressants, psychotropic drugs, immunosuppressants, intestinal microecological agents, laxatives, or antidiarrheal drugs for more than 3 days within 2 weeks of enrollment; (2) have serious systemic diseases (such as abnormal liver and kidney function and abnormal heart and lung function); (3) have a history of gastrointestinal or abdominal surgery.

This study was approved by the Ethics Committee of Huashan Hospital, ethics number: (2019), Linshen No. (471). All subjects signed an informed consent form.

### 2.2. Exhaled H_2_S Determination

The breath test instrument is the Nanocoulomb breath analyzer DA6000 (Wuxi Sunvou Medical Electronics Co., Ltd., Wuxi, China). The measured concentration of hydrogen sulfide is one part per billion (part per billion, ppb). The instrument uses the electrochemical H_2_S gas sensor. The lower limit of H_2_S concentration detection is 3 ppb, detection error is ±3 ppb or 10%, and detection range is 0–3000 ppb. The instrument is designed to operate in accordance with the sampling techniques in 2005 ATS/ERS [10] and the 2019 Rome Consensus [11] as well as 2017 ERS technical standards for exhaled biomarkers [12]. In order to ensure the reliability and repeatability of breath sampling, the flow rate, expiratory pressure, and duration of exhalation are set at 50 ml/s, 10 cm H_2_O, and 10 s, respectively. In order to eliminate the influence of H_2_S in the environment, the subject first inhales through the H_2_S filter and then exhales according to the set flow rate, expiratory pressure, and duration of exhalation. Standard gas of 50 and 200 ppb H_2_S/N_2_ provided by the manufacturer is used for calibration before the test every day in order to ensure the accuracy of the detection.

Taking into account the impact of oral H_2_S on the detection of H_2_S in the digestive tract, nasal exhalation is adopted to determine H_2_S concentration besides oral exhalation. Results of nasal and exhaled determination are then compared and analyzed.

The detailed operation process is as follows. (1) Subject preparation: all subjects only eat rice and meat the day before the test, must fast 12 h before the test, and avoid workout and smoking on the day of the test. (2) Operation process: gargle before the test; during exhaled determination, the subject uses a disposable filter to wrap the lips tightly. After inhaling through the filter, hold the breath for 15 s and then exhale with some force. Coordinate the exhalation rhythm through animation software. The analyzer will automatically collect the end-expiratory air. During the nasal exhalation measurement, the subject uses the disposable nasal filter to align with the single test nostril and then holds breath after 15 s natural inhalation. As you block opposite nostril with your hand, exhale with a certain strength and coordinate the exhalation rhythm through the animation software. The analyzer will automatically collect the end-expiratory gas. After the above collection process is completed, the analyzer will automatically analyze the exhaled gas and displays the result immediately.

### 2.3. Statistical Method

SPSS 15.0 statistical software was used for data analysis. Normally, distributed data in the measurement data are represented by the mean ± standard deviation, and nonnormally distributed data are represented by the median (interquartile range). *P* < 0.05 is considered statistically different. *P* < 0.01 is considered significantly different.

## 3. Result

### 3.1. Rank-Sum Test Result

The ratio of male and female is 39 : 31, and the average age is 61 ± 13 in the observation group. The ratio of male and female is 34 : 32, and the average age is 56 ± 14 in the control group. Two groups' baseline values are comparable, and there is no significant difference (*P* > 0.05).

**Figure 1:**
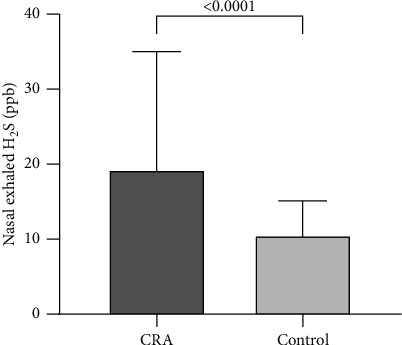
Nasal exhaled H_2_S comparison between the observation group and control group.

The nasal exhalation indicator H_2_S in 70 cases of colorectal adenoma and 66 cases of the control group were analyzed by the rank-sum test. The results showed that (1) the nasal exhaled H_2_S in the observation group was significantly different from that in the control group (*P* < 0.05) (Table 1, Figure 1).

**Figure 2:**
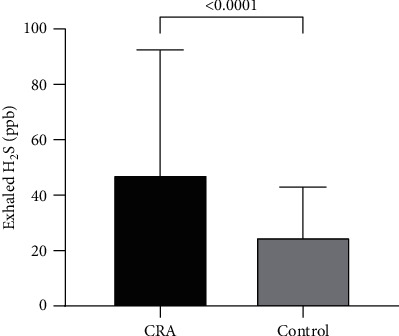
Exhaled H_2_S comparison between two groups.

The exhaled indicator (H_2_S) in 70 cases of colorectal adenoma and 66 cases of the control group was analyzed by the rank-sum test. The results showed that there was a significant difference in exhaled H_2_S between the CRA group and Huashan control group (*P*  ≤  0.001) (Table 1 and Figure 2).

### 3.2. AUC Curve Analysis

**Figure 3:**
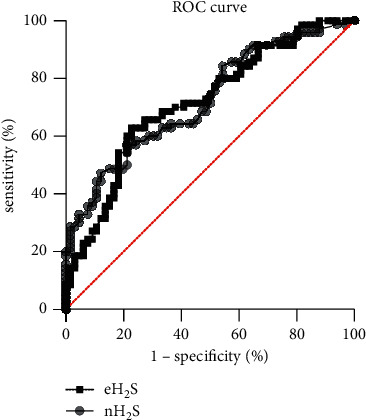
ROC curve of nasal exhaled H_2_S (nH_2_S) and oral exhaled H_2_S (eH_2_S).

Diagnostic predictive value of exhaled H_2_S in colorectal adenoma: ROC curve analysis of the nasal exhaled H_2_S of the two groups showed that the AUC was 0.724, which had a diagnostic value. When the nasal exhaled H_2_S >13.3 ppb, the sensitivity and specificity of predicting CRA were 57% and 78%, respectively (Figure 3). ROC curve analysis of exhaled H_2_S of the two groups showed that the AUC was 0.716, which had a certain diagnostic value (Figure 3). When exhaled H_2_S >28.8 ppb, the sensitivity for predicting CRA was 63% and the specificity was 77%. Above analysis suggested that exhaled determination was better than nasal exhaled determination.

## 4. Discussion

CRA is recognized as precancerous lesion of CRC, and CRA has common pathophysiological basis as CRC. The increase of hydrogen sulfide production is one of those typical pathophysiological characters. Although, H_2_S in the intestine may play a two-way role in the occurrence and development process of CRA/CRC [13], in which the overall trend of H_2_S production is rising amongst people with CRA based on current research. On the one hand, the synthesis of endogenous H_2_S goes up in tumor cells. On the other hand, gut microbiota metabolism shifts to prone to H_2_S production during tumor genesis.

Currently, cystathionine beta-synthase CBS, cystathionine beta-synthase CSE, and 3-MST are the three main enzymes that endogenously synthesize H_2_S. These enzymes are all related to the occurrence and development of malignant tumors [13]. Some studies have found that CBS increases significantly in CRA/CRC [14–16]. According to Phillips et al. [16], the expression of CBS in intestinal adenoma epithelium was upregulated during the development of CRA, leading to increased H_2_S. CBS expression was related to the upregulation of NF-*κ*B, K-RAS, and *p*53 signaling pathways as well. H_2_S further promoted glycolysis of adenoma epithelium and production of ATP, thereby boosting the abnormal division and proliferation of adenoma epithelium and accelerating CRA development.

Besides endogenous synthesis, H_2_S can derive from gut microbial metabolism [17]. Flannigan et al. [18] demonstrated that more than half H_2_S was generated by intestinal microbiome. Shen et al. [19] also illustrated that the proportion of H_2_S produced by intestinal microbiota accounted for the majority of H_2_S produced by human body according to an experimental study on sterile mice and bacterial colonized mice. The H_2_S-producing intestinal microbiome is divided into two main types. The first type of bacteria generates H_2_S by metabolizing sulfur-containing amino acids (similar to the CBS pathway of tissue cells), including *Fusobacterium*, *Clostridium*, *Escherichia coli*, *Salmonella*, *Klebsiella*, *Streptococcus*, *Desulfovibrio*, and some *Enterobacter*. Another type of bacteria produces H_2_S through the sulfate metabolism, mainly *Desulfovibrio* [7]. Vacante [7] discovered that Biliophilus and *Desulfovibrio* increased significantly (control = 547) in the flora of tumor tissue and peripheral intestinal epithelium among CRA patients (*n* = 233). Moreover, the increase of Biliophilus and *Desulfovibrio* were obviously related to the increase of metabolite-H_2_S, which had the potential value for diagnosing CRA as a characteristic change. In the follow-up studies, the author found that the H_2_S ion current test for colon cancer epithelium, peripheral epithelium, and distal epithelium showed a high-to-low change, which was statistically significant among CRC patients (*n* = 106). Meanwhile, some intestinal bacteria associated with H_2_S change became different, such as *Clostridium sclerotium*, *Clostridium perfringens*, nematodes gingivalis, and *Bacteroidesfragilis* [20]. These findings clearly indicated that H_2_S strains derived from CRA were closely related to the progression of CRC.

After clarifying the potential value of H_2_S in the diagnosis of CRA, many scholars explored the specific methods of H_2_S application in CRA/CRC as well. These studies focused primarily on the detection of H_2_S in human peripheral blood and feces, using methods such as methylene blue, monobromodimarane, S2-electrode ion detection, and mass spectrometry. However, methylene blue, monobromodimarane, and S2-electrode ion detection methods are susceptible to pH changes, so measured results may have large fluctuations. In addition, H_2_S is relatively unstable and can be converted to methyl mercaptan and dimethyl disulphide in the body, which makes it difficult to draw high repeatable conclusions. Mass spectrometry is complicated and expensive and is rarely used. Therefore, a high-precision electrochemical sensor was adopted in this study to determine exhaled H_2_S content among CRA patient population by the point-of-care test. The advantages of exhaled determination are as follows: (1) the pH and temperature in the circulating blood are relatively stable, so H_2_S is less affected, and the concentration of free H_2_S is relatively stable; (2) the POCT avoids H_2_S being oxidized or converted to other forms; (3) holding the breath for 15 s ensures the full exchange of circulated gas molecules in the alveoli, which can truly reflect H_2_S concentration; (4) the accuracy of the measurement is ppb level (ambient gas detection is usually ppm level), which is more sensitive to indicate the degree of H_2_S change.

In this study, exhaled H_2_S of the CRA group was significantly higher than that of the control group, which was statistically different. Both two sampling methods of oral exhalation (AUC 0.716) and nasal exhalation (AUC 0.724) obtained from the above results had good diagnostic value based on ROC analysis, confirming the accuracy of exhaled H_2_S determination for CRA screening. Hampton [21] reported that the oral flora was associated with colonic bacterial colonization. For example, *Fusobacterium nucleatum* was one of the common facultative anaerobes in the oral cavity, but it was rare to be seen in the healthy people's intestine. The association between oral and colonic flora may be the key to maintaining the same trend for oral exhalation and nasal exhalation. This conclusion was in line with conclusions of previous studies [4, 6, 9], which reflected that the overall H_2_S was at a high level within the CRA population. From the realm of the physiological mechanism, exhaled H_2_S is derived from the total H_2_S excreted from the body through the alveolar gas exchange. A portion of total H_2_S is synthesized by the CRA tissue epithelium and the rest H_2_S is produced by intestinal flora. Besides, change tendency of H_2_S from the two sources is determined, which is conducive to the consistent judgment of the results.

In the process of exhalation, the influence of H_2_S from other sites on the results mainly included the sources of upper airway (nasal cavity) [22], lower airway (lung tissue and trachea) [23], and oral cavity [24]. How to eliminate the effect is one of the key considerations. In terms of H_2_S source of upper airway (nasal cavity), the median of H_2_S measured by nasal ventilation was 2 ppb. Nasal H_2_S further reduced in patients with seasonal allergic rhinitis compared with the nonallergic rhinitis group according to the study of Li et al. [22]. For the H_2_S source of lower airway, Zhang et al. [23, 25] demonstrated that exhaled H_2_S concentration in the lower airway among some patients with either asthma or COPD increased compared with the control group. However, increase was inversely proportional to the number of eosinophils. From the perspective of inflammatory cell types and exhaled H_2_S concentration, oligogranulocytic > neutrophilic > eosinophilic granulocytosis [25]. Even the more obvious inflammatory cell infiltration in the airway (regardless of nasal or tracheal origin) was, the less H_2_S was produced. In other words, there was a negative correlation between the granulocytic infiltration and H_2_S production. Therefore, based on the above research results, it was concluded that airway inflammation had little impact on the total exhaled H_2_S volume. Oral cavity is the third part of H_2_S source. Due to the presence of oral bacteria, Pysanenko et al. [24] found that H_2_S measured orally was significantly higher than nasally, while Dryahina suggested that using nasal exhaled H_2_S determination could better reflect the H_2_S from the intestinal tract. Therefore, we added nasal exhaled H_2_S determination on the basis of oral exhaled H_2_S determination, which meant that the exhalation site was replaced with the nose. Nevertheless, the breath-holding time, flow rate, and end-expiratory sampling method remained unchanged in order to eliminate the influence of the oral cavity, which further verified the predictive value of exhaled H_2_S on CRA. Results revealed that the nasal exhaled H_2_S (mean: 19.32 ± 15.71 ppb) in the CRA group was significantly higher than that in the control group (mean: 25.00 ± 17.94 ppb). In the ideal model, the nasally exhaled sampling method that excludes the influence of the oral cavity should have a better diagnostic value. Although, in this study, the ROC of the nasal exhaled sampling was slightly higher than that of the oral exhaled sampling (nasal exhaled AUC 0.724 vs. oral exhaled AUC 0.716), there is no significance between two AUCs (*P*=0.86).

However, study design had some disadvantages. For example, fasting exhaled H_2_S was a reflection of the total H_2_S in the human body. Some factors may limit the diagnostic value of fasting H_2_S on CRA, such as whether H_2_S derived from adenoma tissue or other human tissues was affected by other factors and whether intestinal flora metabolism was affected by food, drugs, and H_2_S peaks. Banik et al. [26] used fasting H_2_S as a baseline value in an IBS study. The exhaled H_2_S determination was performed 45 min after oral administration of the substrate lactulose. Subtract the baseline value from the test value as △H_2_S to determine whether IBS merges SIBO. This research method was to optimize the exhalation method under the condition of limiting the microbiota metabolism. How to choose a better preparation and sampling method for detecting CRA patients remained to be further explored in order to achieve an optimal diagnostic value.

In conclusion, CRA screening is essential for detecting early cancer of colon tumors. The study used exhaled H_2_S determination that was a noninvasive, accurate, and innovative method. Exhaled H_2_S determination provides a potential CRA inspection method and compensates shortcomings of traditional endoscopy and plasma laboratory testing, so it is worthy of further research and generalization.

## Figures and Tables

**Table 1:** Results of exhaled determination in the observation group and control group.

	Observation group	Control group
Number of people (male/female)	70 (39/31)	66 (34/32)
Age	61 ± 13	56 ± 14
Nasal exhaled H_2_S (ppb)	19.32 ± 15.71^*∗∗*^	10.59 ± 4.53
Exhaled H_2_S (ppb)	47.47 ± 44.95^*∗∗*^	25.00 ± 17.94

^*∗∗*^Statistically significant difference between groups, *P* < 0.01.

## Data Availability

The data used to support the findings of this study are currently under embargo while the research findings are commercialized. Requests for data, 12 months after publication of this article, will be considered by the corresponding author.
